# Automated tracing of helical assemblies from electron cryo-micrographs

**DOI:** 10.1016/j.jsb.2017.11.013

**Published:** 2018-04

**Authors:** Stefan T. Huber, Tanja Kuhm, Carsten Sachse

**Affiliations:** European Molecular Biology Laboratory (EMBL), Structural and Computational Biology Unit, Meyerhofstraße 1, 69117 Heidelberg, Germany

**Keywords:** Electron cryo-microscopy, Helical assemblies, Automated particle detection, Image pattern recognition, Persistence length

## Abstract

Structure determination of helical specimens commonly requires datasets from thousands of micrographs often obtained by automated cryo-EM data acquisition. Interactive tracing of helical assemblies from such a number of micrographs is labor-intense and time-consuming. Here, we introduce an automated tracing tool MicHelixTrace that precisely locates helix traces from micrographs of rigid as well as very flexible helical assemblies with small numbers of false positives. The computer program is fast and has low computational requirements. In addition to helix coordinates required for a subsequent helical reconstruction work-flow, we determine the persistence length of the polymer ensemble. This information provides a useful measure to characterize mechanical properties of helical assemblies and to evaluate the potential for high-resolution structure determination.

## 1 Introduction

Visualization of biological macromolecules by electron cryo-microscopy (cryo-EM) is one of the best suited methods to study the three-dimensional (3D) structure of large assemblies. Highly symmetrical assemblies, in particular helical structures, have been critical to establish methodology of 3D image reconstruction (DeRosier and Klug, 1968) as well as for further developments for high-resolution structure determination (Beroukhim and Unwin, 1997, Fromm et al., 2015, Ge and Zhou, 2011, Sachse et al., 2007, Yonekura et al., 2003). In addition, large helical assemblies constitute a fundamental architectural building principle in biology found in cytoskeletal proteins, viral capsids, enzymes, amyloid fibrils, membrane-remodeling and signaling complexes (Frost et al., 2008, Hirose et al., 1996, Lynch et al., 2017, Moore et al., 1970, Sachse et al., 2008, Wu et al., 2014), recently reviewed (Sachse, 2015). As new structures of helical assemblies are determined, new functional roles are being discovered in various processes of the cell (Ciuffa et al., 2015, McCullough et al., 2015). With improved hardware and software near-atomic resolution structures of these assemblies are increasingly common and critical to reveal the structural basis of the underlying assembly mechanism.

One of the main reasons for the improved performance of cryo-EM structure determination were due to hardware developments. First, direct electron detectors with improved detective quantum efficiency (DQE) and frame readout gave rise to images of much improved quality (McMullan et al., 2009, McMullan et al., 2016). Second, microscopes of improved stability with software-assisted automation are now commonly used to generate 1000 s of micrographs from a single sample (Biyani et al., 2017, Mastronarde, 2005, Suloway et al., 2005). Another important advance comes from software improvements and high-performance computing that have reduced user interference and made large-scale image processing on high-performance computing architectures feasible over the last decades (Desfosses et al., 2014, Frank et al., 1996, Grigorieff, 2007, Ludtke et al., 1999, Scheres, 2012, Sorzano et al., 2004, Tang et al., 2007). In order to obtain near-atomic resolution in most cases 100,000 s of asymmetric molecular units need to be analyzed before averaging. Whereas for single particles a plenitude of semi-automated or fully automated procedures exist, for helical specimens most of the specimens are digitally excised by a user-guided interactive cropping procedure (Ludtke et al., 1999, Tang et al., 2007) before helices are being segmented into a stack of single particles. Although this process generates helical data sets of high confidence, it is labor-intense and time-consuming and can often take days for large data sets before image processing can be initiated.

The need for automated particle detection on micrographs from negative stain had been realized in the early 1980s (Frank and Wagenknecht, 1983) and since then many approaches for single particle detection have been introduced. Particle detection from cryo-micrographs is even more demanding due to the poorer signal-to-noise ratio. Multiple algorithms have been put forward to address this technical challenge. The proposed approaches rely on principles of template matching by reference-based cross-correlation (Roseman, 2003), pattern recognition (Zhu et al., 2003), edge detection (Harauz and Fong-Lochovsky, 1989) and other types of intensity measures reviewed by Glaeser and colleagues (Nicholson and Glaeser, 2001). More recently, deep learning algorithms based on neuronal networks have been added to the repertoire of techniques used for particle detection (Wang et al., 2016, Zhu et al., 2017). The principle goal of the particle picking workflow can be summarized as follows: to precisely locate the particle while avoiding to recognize noise or contaminants. In most cases, additional pruning is required either by a human operator or other means. For this reason, many programs are semi-automated such that they include a user GUI and require a final step of human intervention (Frank et al., 1996, Ludtke et al., 1999, Scheres, 2015, Tang et al., 2007).

For helical specimens, there are very few automated approaches known to date suited for fully automated or semi-automated selection of filamentous or elongated helical assemblies. One of the earliest approaches was developed by combining near and far-to-focus images to trace the central helical axis using the helix contours segmented by a Canny edge detector (Zhu et al., 2001). Although particularly useful for images of wide and rigid specimens such as tobacco mosaic virus (TMV), such approaches are operationally challenging due to dual image acquisition and it is more difficult to recognize thinner filaments with less molecular mass. In the past, the majority of helical assemblies were still excised interactively as relatively few high-quality images were sufficient to compute 3D maps of helical assemblies (Yonekura et al., 2003). With the introduction of single-particle image processing to helical reconstruction (Egelman, 2000, Jiménez et al., 1999), more flexible and heterogeneous helical structures became amenable to 3D image reconstruction. Therefore, classification methods originally developed for single-particle image processing algorithms could be directly applied to helical structures (Behrmann et al., 2012, Desfosses et al., 2014, Wang et al., 2006). More recently, the maximum-likelihood based RELION software (Scheres, 2012) has been adapted to work with helical structures. Based on the single-particle framework an adapted semi-automated helix detection workflow has become available (He and Scheres, 2017). Once the helical axis has been determined on the micrograph, the segments can be extracted and subjected to the respective image processing pipeline. In addition, traces of elongated specimens can also be used to assess material properties on the rigidity and flexibility of the examined structures (Sachse et al., 2010, Wang et al., 2002). For high-resolution and lower-level structural characterization a fast implementation is essential to reliably trace elongated assemblies while minimizing human intervention.

In the current manuscript, we introduce a robust micrograph-based helix tracing (MicHelixTrace) algorithm that automatically detects helical specimens based on a reference image. Computational cost is minimized by a reduced search separating estimation of rotational and translational parameters for helical specimens in Fourier and real space domain respectively. The resulting cross-correlation map is thresholded and helix coordinates are extracted from binarized skeletons. We demonstrate that the approach is successful for rigid assemblies of TMV, thin cytoskeletal ParM filaments as well as flexible p62-PB1 filaments. In addition to helix coordinates, the introduced automated tracing approach also determines fundamental material properties in the form of persistence length for helical structures. The automated helix detection algorithm MicHelixTrace provides a computationally fast implementation for faithful tracing of helical assemblies from electron cryo-micrographs with minimized human intervention.

## 2 Principle of the approach

**Fig. 1:**
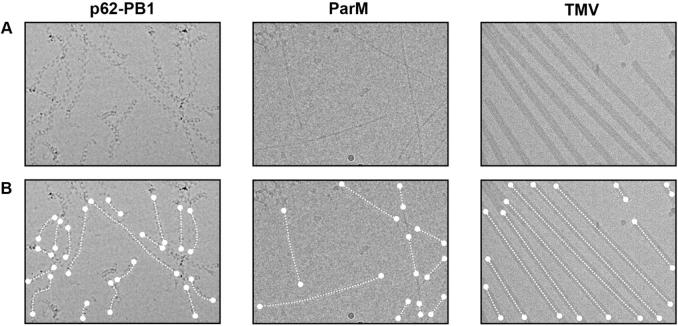
Typical micrographs of three helical specimens with varying degree of straightness. (A) Three micrographs of helical specimens p62-PB1 (left), ParM (center) and tobacco mosaic virus (TMV) (right), respectively. (B) Superimposed traces of respective helical specimens. Each helix trace contains a starting and an end coordinate (dot) that are connected by a set of equidistant segment coordinates forming a trace (dashed line). The trace approximates a straight line for very rigid helical assemblies like TMV.

**Fig. 2:**
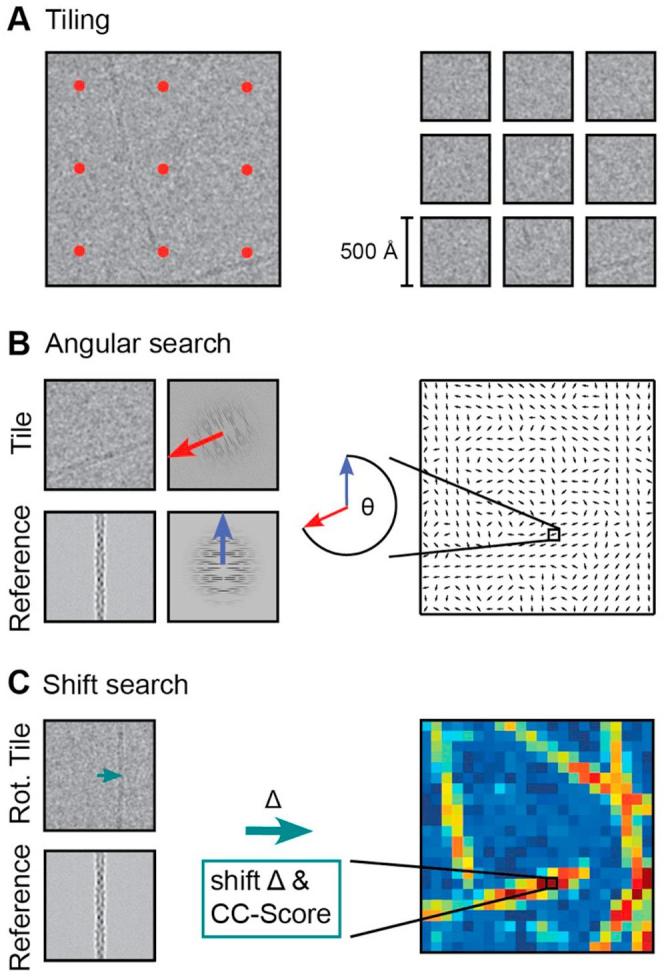
Tile-based computation of local correlation function of the micrograph using a helix reference image. (A) Tiling of the micrograph (left) into overlapping squares of 350–500 Å in dimension (right). (B) Determination of in-plane tile rotation *θ* with respect to reference helix by angular search between power spectra of reference and tiles (left). The step yields a tile map that contains the assigned rotation value *θ* as summarized in the vector plot (right). (C) Determination of distance (shift) to helix axis *Δ* by cross correlating rotated tile and reference helix image (left). The step yields a tile map that contains the shift *Δ* and cross-correlation (cc) score (right).

One of the most successful approaches to automatically detect single particles from a reference image is based on the local cross-correlation function (Chen and Grigorieff, 2007, Roseman, 2003, Scheres, 2015), which yields the x and y positions of the particles on the micrograph. In contrast, helical assemblies are long entities that extend continuously for more than 500 Å in one direction. Consequently, their location description can be reduced to a trace with start and end coordinate pairs only when they resemble a line or by a set of equidistant segment coordinates when the helical axis deviates from a straight line (Fig. 1). Computing complete local correlation functions for every possible location on the micrograph is computationally demanding. In order to estimate end, start and segment coordinates of a continuous helix, computing a much smaller subset of the local correlation function is sufficient. For this purpose, we subdivided the micrograph into tiles with 80% overlap along x and y to match the provided reference image (Fig. 2A). Tiles of 350–500 Å dimension bear the advantage that the helical axis can be approximated as a line within this window even though for larger dimensions the polymer deviates from a straight line. The path of the helix within the tile can be explained by the in-plane angle *θ* and the normal distance or shift relative to the tile center *Δ* (Fig. 2B/C). To determine *θ* and *Δ*, an exhaustive search could be employed to further localize the helix within the window using a multi-dimensional cross-correlation (cc) function. In order to cut down on the computational cost of this search, we separate the in-plane rotation *θ* from the positional *Δ* search. Due to their symmetry, helical specimens possess layer lines in the Fourier domain. As the amplitude spectrum is invariant to translation of the object in real space, we can reliably determine the rotation *θ* by a rotational correlation function directly from the power spectra of the tile and the reference. According to the determined helix angle, we then rotate the tile and compute only one correlation function to determine the shift relative to the tile center *Δ* including a cc-score. As a result of the procedure, the *θ/Δ* pair and a normalized cc-score is obtained for every tile.

**Fig. 3:**
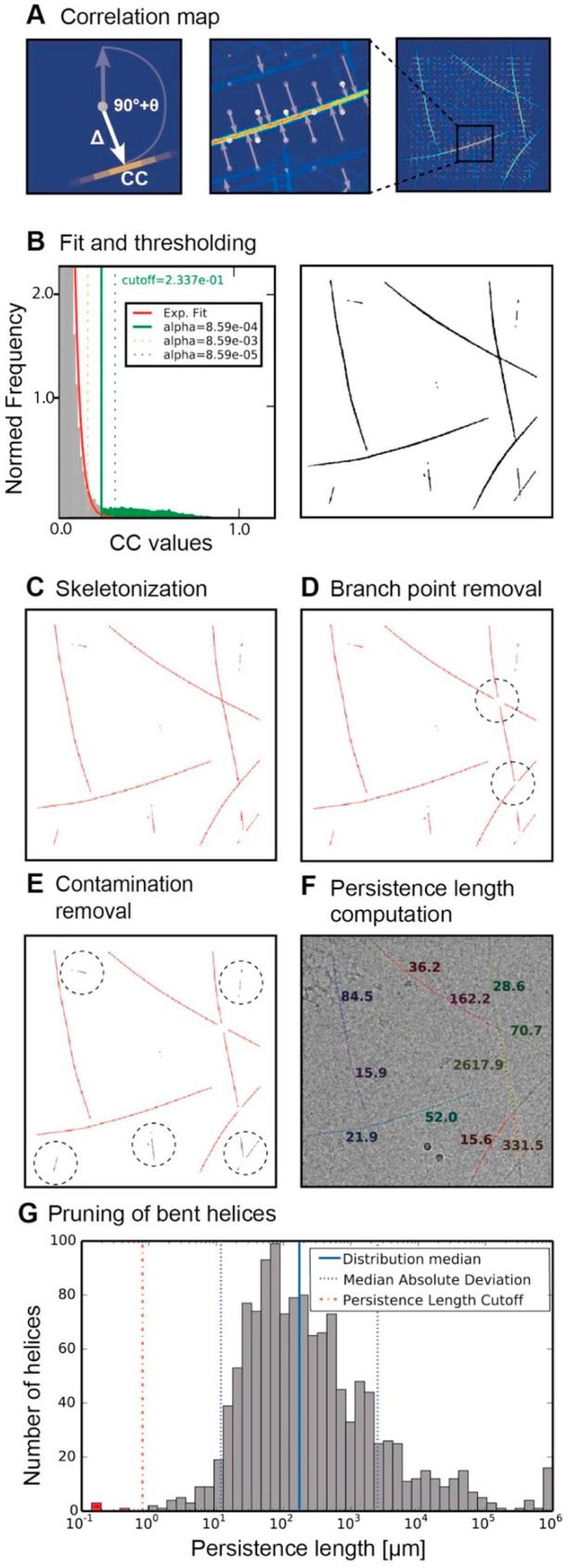
Extraction of helix coordinates from the cross-correlation map. (A) Position mapping of helix traces into micrograph by combining tile rotation *θ*, shift *Δ* and cc-score. (B) Histogram of correlation map values contains predominantly low random correlations and some high correlation values from helices. The integral of the histogram bins is normalized to 1. The histogram can be fit by an exponential distribution (red line) corresponding to the background of low correlations. The α-threshold can be used as a cutoff value (green line) to separate the tail (green bins) from the background (grey bins). Pixel values beyond the α-threshold correspond to helical traces (right). (C) Binarized map is skeletonized to one-pixel wide traces for further processing. (D) Areas of overlapping traces are found by branch point detection (dashed circles) and erased. (E) Traces shorter than the minimum helix length (dashed circles and blue) are excluded resulting in effective contamination removal. (F) Remaining traces are extracted and fitted by polynomials to present helical traces in coordinates. Persistence length for traces is computed. (G) Histogram of persistence lengths (log scale) from helix population of entire micrograph set. Determination of median enables pruning of highly bent outliers for helices that have persistence lengths of two standard deviations (computed as median absolute deviation) below the mean.

**Supplementary Fig. 1:**
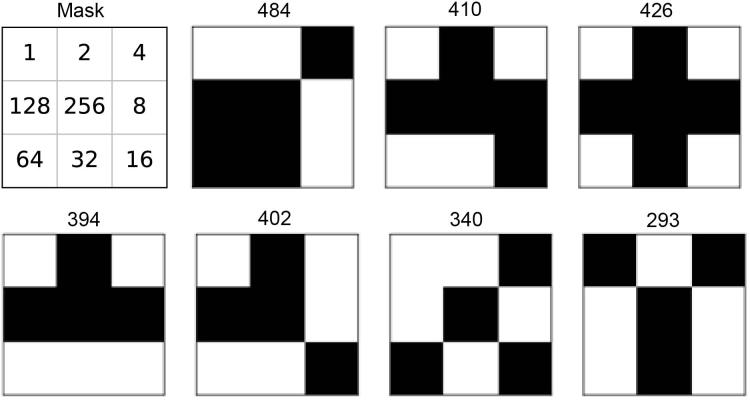
Branch point detection in skeletonized binary map. Mask [[1, 2, 4], [128, 256, 8], [64, 32, 16]] containing power of 2 elements (upper left), and seven unique pixel configurations within a 3 × 3 neighborhood, which correspond to branch points of helix overlap. The integer value on top represents the response of the convolution of a 3 × 3 neighborhood with the mask.

Based on the determined *θ* and *Δ* values of each tile, cc-scores can be mapped back on the micrograph with much higher precision than the initial coarse tiling (Fig. 3A). The resulting micrograph map exhibits high correlation for paths where the reference is present and shows small correlations where the reference is absent. In the next step, the cross-correlation map requires thresholding to reliably extract the helical paths. For this purpose, we evaluated the background correlation distribution in a histogram and found that it follows an exponential distribution for areas devoid of helices (Fig. 3B). Only few high correlation values represent the paths of the helices, which are above the null distribution visible in a tail of the histogram. After fitting the null distribution and estimation of the tail threshold, we binarize the correlation map. Subsequently, the paths are skeletonized to single pixels by a morphological thinning procedure (Fig. 3C) (Lam et al., 1992). Overlapping paths can be identified as branch points by a feature-specific response filter (Olsen et al., 2011). Once thinned to single pixel paths, branch points can only occur in a limited set of pixel configurations that can be located by a precomputed convolution response (Supplementary Fig. 1). Subsequently, overlapping helices are split into separate helices (Fig. 3D). Finally, helices shorter than a user-defined minimum helix length parameter are discarded as they often correspond to contaminations and non-helical protein, for example aggregates (Fig. 3E). Longer helices are broken up into pieces of comparable size and the remaining traces are fitted by a 1st to 3rd order polynomial function to yield a set of equidistant segment coordinates including the start and end points of the helix (Fig. 3F). In a final pruning step, we evaluate the determined traces with respect to straightness of the helix population from the micrograph set. As thermal helix trace fluctuation is determined by their inherent material properties, helices outside the expected distribution should not correspond to the targeted assemblies. In fact, they are often kinked helices unsuitable for helical reconstruction or represent false positive hits, e.g. contamination perhaps of elongated shape, which is erroneously detected as a helix (Fig. 3G). After the final straightness analysis, we can eliminate those helices that do not match the expected straightness range.

## 3 Implementation

### 3.1 Preprocessing of the micrograph into tiles

In order to locate the helical axis of the filament within the micrograph, the micrograph is normalized and commonly binned by a user-defined binning factor, e.g. 8-fold to decrease computational costs. To remove grey-scale ramps within the micrograph a Gaussian high-pass filter with half-maximum intensity at 4% of Nyquist is applied. Extreme intensity features such as gold particles, hexagonal ice crystals or ice droplets are reliably excluded by fitting a normal distribution to the micrograph’s pixel intensities using the image median and the MAD (median absolute deviation) rather than mean and standard deviation as the former is less sensitive to outliers (Leys et al., 2013). Subsequently, the pixel intensities are transformed to *p*-values and pixels with a value less than 0.001, corresponding to very dark pixels are set to match the median. The micrograph is windowed into overlapping tiles using the input parameters ‘power tile size’ and ‘tile overlap’. In all test cases, we successfully used tile sizes between 350 and 500 Å and an overlap of 80% along x and y corresponding to an effective step size of 70–100 Å between tiles, respectively. The step size determines how finely the cross-correlation grid is sampled on the micrograph and therefore how well adjacent helices can be discriminated. The used step sizes are in the range of the half-widths of the investigated helical assemblies. Every tile is multiplied by a circular Gaussian real-space mask with a standard deviation of 1.41 times the tile step size to improve separation of helices when they are closely packed in one tile.

### 3.2 Reference helix

The algorithm requires a reference helix image as input, with the helix axis aligned vertically. Contrast-transfer function correction is not required as we found that for helix tracing high-frequency information is not necessary. For best performance, we recommend using a class average generated from a small amount of interactively traced helices in a subset of micrographs, e.g. using SEGMENTCLASS from the SPRING package (Desfosses et al., 2014). Although providing the class average makes subsequent automated tracing more robust, in the case of high contrast specimens, like for TMV, a single manually traced helix was found to be sufficient to act as a reference, e.g. obtained by EMAN2’s E2HELIXBOXER (Tang et al., 2007).

### 3.3 Tile-based angle and shift determination

In order to determine the angle of the helical axis with respect to the image rows in the tile, we rotationally align the power spectrum of the tile against the power spectrum of the reference helix. As a result, we obtain the best correlating angle *θ* (Fig. 2A). After applying the angle to the tile, we now compute the cross-correlation function between the tile and the reference helix (Fig. 2B). From the maximum cc-value, we obtain a corresponding *x/y* vector that is pointing to the position where the helical axis is located in the tile. From this shift vector, we extract the component normal to the helix axis called *Δ*.

### 3.4 Generation of continuous cross-correlation map

For every tile, we obtain a value for angle *θ*, shift *Δ* and an associated cc-score. By converting *θ* and *Δ* of the individual tiles into absolute positions of a single map, we derive the location of the helical paths within the micrograph. For every tile, we draw a line of the cc-intensity into the adjusted respective location of the helix. The line intensity has a Gaussian falloff with *σ* = 2 ∗ step size between tiles from the determined center to better take into account helix start and end positions. Tiles with no helix will have a low cc-score and a random angle *θ* and shift *Δ* and will thus not lead to strong features in the localization map. Tiles containing a helix will contribute additively to a line making up a continuous helical path in the cross-correlation map.

### 3.5 Thresholding of the cross-correlation map

We take all non-zero values from the correlation map above. The noise correlations follow an exponential falloff whereas correlation values of helix paths are present in the tail above the null distribution. The exponential null distribution can be described as:

(1) $$f_{\lambda }(x)=\lambda *\exp\left[-\frac{x}{\lambda }\right]$$

We estimate *λ* by calculating the median of the cc-values:

(2) $$\lambda =\frac{\mathrm{median}}{\ln(2)}$$

Even in the presence of high correlations, Eq. (2) approximates the null distribution well, as the majority of values belong to the background and the median is robust with respect to outliers. We transform the correlation map values to *p*-values, corresponding to the probability that this or a more extreme value occurs under the fitted exponential null distribution. The user specifies a significance level α from when on to regard a *p*-value as significant. A typical value α = 0.001 can be interpreted as a 0.1% risk of finding false positive traces where no actual helix exists. This significance value is used to threshold the cross-correlation map. The higher the α-value used for thresholding of the cross-correlation map, the more false positive helix traces will appear in the resulting binarized map.

### 3.6 Skeletonizing and splitting of overlapping helices

In order to precisely estimate the helix path and remove overlapping segments from multiple crossing helices, the binarized traces require to be skeletonized. The procedure removes pixels on the boundaries of objects to make them one pixel thin without separating connected components. The matlab-function bwmorph was ported to python to skeletonize the binary image from above. To avoid overlapping helices, we identify branch points by analyzing all 3 × 3 tiles in the skeletonized binary image. Only a limited set of seven unique binary 3 × 3 tiles were identified that make up possible branch points. All of those branch point tiles are also considered in 4 different rotations and in their mirrored versions. We use a unique 3 × 3 mask that is encoding values with 2^n^, *n* = 1, 2, … 8 for each position (Supplementary Fig. 1). When convolving the skeletonized binary image with this mask, every pixel of this response filter is confined to integer values between 0 and 511. Using precomputed responses of the limited set of branch points (Supplementary Fig. 1), we identify the locations of branch points. Once located, we remove the branch points by erasing a circular area with helix diameter from the skeletonized binary image.

### 3.7 Contamination removal and polynomial fitting of traces

From the resulting binary image, we extract connected components in the form of coordinates and calculate their contour length. As noise and contamination can still give rise to short paths in the thresholded correlation map, the user provides a minimum helix length to be included in the tracing (Fig. 3E). In addition, a second parameter will be required for the maximum helix length, which will separate longer helices in multiple independent traces if required. The remaining connected traces are assumed to correspond to individual helices in the micrograph. The pixel coordinates contained in every connected component are used to perform a polynomial fit using Numpy’s polyfit function (Fig. 3F). Keeping helix lengths within the described boundaries throughout the data set will impose a comparable stiffness restraint on the polynomial fitting of the traces. We found when helix lengths remain between 500 and 2000 Å, a simple quadratic fit is sufficient also to describe the paths of flexible polymers.

### 3.8 Pruning based on expected straightness

In order to remove kinked or unrealistically bent helices, we determine a straightness measure for each identified helix trace. Based on the isolated contours, we can directly compute the flexibility parameter *λ* using the approximation formula (Trachtenberg and Hammel, 2010) by taking into account the end-to-end distance *R* with the contour length *L* of the helical path:

(3) $$\lambda =\frac{-\ln\left[2{\left(\frac{R}{L}\right)}^{2}-1\right]}{L}$$

The flexibility parameter *λ* is related to the persistence length measure *p* of the helix by the following formula:

(4) $$p=\frac{1}{\lambda }$$

Stiffer polymers give rise to higher values of persistence lengths whereas lower values correspond to more flexible specimens. After the traces from the entire micrograph set have been determined, the helix population will be analyzed with respect to the determined persistence lengths. The histograms of *p* follow a log-normal distribution (Fig. 3G). For pruning, traces that have persistence lengths of two standard deviations below the mean are being discarded. To increase robustness, we estimate mean and standard deviation with distribution median and MAD (Leys et al., 2013). The user has to specify the multiple of the standard deviation to exclude below the mean and thereby removing the most bent helices from the population.

### 3.9 Performance evaluation by interpreting algorithm as binary classifier

We evaluated the performance of the proposed MicHelixTrace procedure as well as helix tracing in RELION by comparing the results with interactively traced helices. Interactive tracing was carried out using E2HELIXBOXER in such a careful way that we consider the picked helix traces as the ground truth. In order to treat the tracing results as a binary classification problem, we converted the traced coordinates to a binary pixel grid. Traces of a micrograph were mapped onto a coarse blank binary grid of 25 Å pixel size, where a pixel value is assigned one if the projected helix path passes through a pixel. As interactive tracing with E2HELIXBOXER yields straight traces and the helical paths do not always lie exactly on the helical axis of the polymer, we allowed an extra margin by binary inflating the grids by the corresponding helix width. Due to missing high-resolution information at the micrograph periphery imposed by the contrast-transfer function, a generous 400 Å margin around the micrograph boundaries is excluded both from ground truth and tracing result grids. An additional ambiguity in the comparison arises from how the operator or the program MicHelixTrace or RELION defines the ends of the helix traces, i.e. whether the end of a trace lies on the end of the polymer or at some constant offset from the end. These helix ends can either be removed in the tracing process or in subsequent segmentation using SPRING. To eliminate this ambiguity from a fair comparison, a circle with radius of two times the helix radius is excluded from both ground truth and tracing result grids. The binary maps of the tracing algorithm and the ground truth helices are compared using the metrics precision *P*, recall *R* and *F1* score (Chinchor, 1992, Van Rijsbergen, 1975).

The precision *P* is defined:

(5) $$P=\frac{\mathrm{\#correct\_traces}}{\mathrm{\#all\_traces\_in\_tracing\_result}}$$

where *#correct_traces* is obtained by counting how many positive grid points of the tracing map coincide with the grid points of the inflated ground truth grid. High *P* values will correspond to small high-quality data sets of correctly identified helices with minimum false positive traces.

The recall *R* is defined:

(6) $$R=\frac{\mathrm{\#found\_traces}}{\mathrm{\#all\_traces\_in\_ground\_truth}}$$

where *#found_traces* is obtained by counting how many positive grid points of the ground truth coincide with the grid points of the inflated tracing result. High *R* values will result in large data sets of found helices at the expense of including more false positives. Consequently, precision *P* and recall *R* are interdependent, i.e. when *P* increases, *R* decreases and vice versa. As a tradeoff between precision *P* and recall *R*, we use the *F1*-score:

(7) $$F1=2\frac{P\cdot R}{P+R}$$

We used Eqs. (5), (6), (7) to assign *P*, *R*, and *F1* values to single micrographs as well as to complete datasets of p62-PB1, ParM and TMV.

### 3.10 Computer programs and parameters

To implement the outlined workflow, we wrote a python software program for micrograph-based helix tracing (MicHelixTrace) that is part of the SPRING software suite (Desfosses et al., 2014). Scientific computation steps are performed by Numpy (Walt et al., 2011), Scipy (Oliphant, 2007) and EMAN2/Sparx (Hohn et al., 2007) libraries. The required input parameters are summarized in Table 1 and listed for three different test cases. Finally, MicHelixTrace outputs either a text file containing coordinate traces in the common EMAN box format or writes out the coordinates in an sqlite3 database file spring.db for further usage inside the SPRING software suite. In addition, we added an option to generate RELION readable ∗box files. In order to compare the performance of MicHelixTrace with RELION, we used RELION 2.05 with the following input parameters: *Minimum Length*: 200 Å, *Shrink* 0.5, *Angular Sampling* 5°. For *Tube Diameter*, we used 180 Å (TMV), 100 Å (p62-PB1) and 80 Å (ParM) and *Maximum Curvature* was set to 0.1 (TMV), 0.4 (ParM) and 0.5 (p62-PB1), respectively. For the ParM and p62-PB1 datasets, five class averages were generated inside RELION to be used as references as suggested in the user manual. For TMV, the 2D classification algorithm converged to a maximum of three occupied classes, so three class averages were used as references. The picking threshold was optimized empirically using a small subset of micrographs.

**Table 1:** Input parameters for three different helical specimens.

	P62-PB1	ParM	TMV
Number of micrographs	148	100	100
Persistence Length in µm	1.27 ± 0.05	33.9 ± 2.9	325 ± 36
Reference			
Reference Type	Class Average	Class Average	Single Helix
Tile size power spectrum in Angstrom	350	500	500
Tile overlap in percent	80	80	80
Binning factor	4	4	8
Estimated helix width in Angstrom	100	80	180
Minimum and maximum helix length	196, 1200	594, 1500	403, 2000
Alpha threshold	0.00113	0.000859	0.117

## 4 Results

### 4.1 Flexibility analysis yields persistence length measures

**Supplementary Fig. 2:**
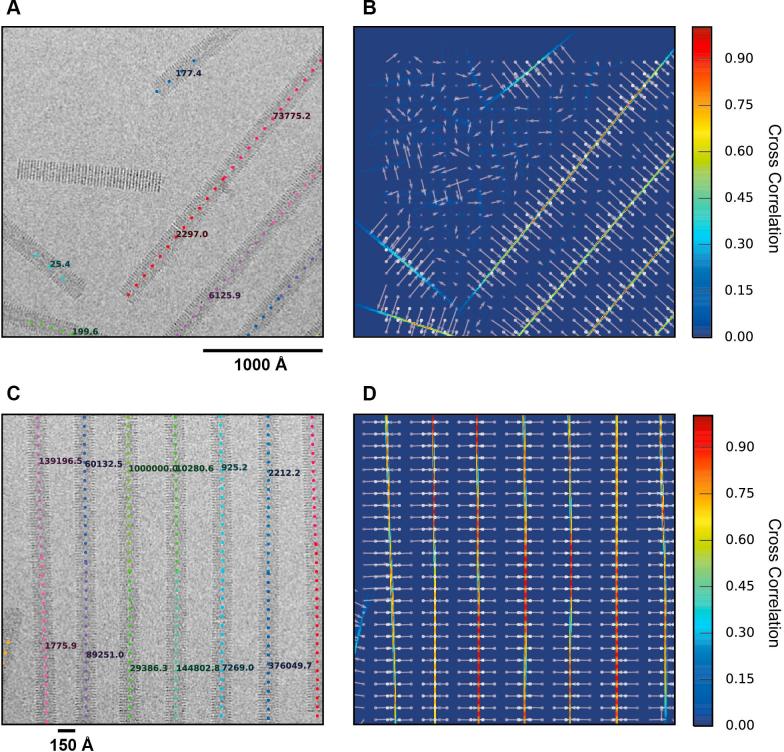
Examples of MicHelixTrace results for TMV. (A) Micrograph containing TMV helical rods and a stacked disc aggregate (center left) is superimposed with detected traces by dots. The stacked disc aggregate is successfully ignored by the tracing algorithm. The number denotes the associated persistence length in µm. (B) The TMV stacked disc aggregate is not apparent in the respective cross-correlation map. (C) Micrograph containing a closely packed raft of TMV rods that were reliably separated by the tracing algorithm. (D) The respective cross-correlation map shows clearly separated helical paths.

We tested the algorithm on three different samples p62-PB1 (Ciuffa et al., 2015), ParM (Bharat et al., 2015a) and TMV (Fromm et al., 2015) that differ significantly in their degree of straightness ranging from flexible to rigid (Fig. 1A). Data sets were recorded on state-of-the-art electron microscopes using a range of common defocus values and dose strategies including two different direct detector types (Table 2). We found that most detected traces overlapped with the true helical assemblies and the program also worked reliably in more difficult recognition scenarios. First, the reference-based tracing routine successfully discriminated different helical structures present within the micrograph as for the case of the TMV sample, where it successfully ignored the minor fraction of stacked disc aggregates (Supplementary Fig. 2A/B). Second, the program also separated closely aligned TMV helices with a distance of 250 Å (Supplementary Fig. 2C/D). Provided that helices resemble the reference, are not overlapping and it is possible to estimate a reliable null distribution of empty micrograph areas, it will be also possible to separate closely packed helices.

**Table 2:** Important imaging parameters of three test data sets.

	P62-PB1	ParM	TMV
Sample preparation	Described in Ciuffa et al. (2015)	Described in Bharat et al. (2015)	Described in Fromm et al. (2015)
Microscope	FEI Titan Krios		
Voltage (kV)	300		
Defocus range (µm)	0.5–4.5	1.0–6.0	0.2–0.6
Dose (e^−^/Å^2^)	56	25–32	35
Pixel size (Å)	1.35	1.07	1.04
Detector	Gatan K2 summit	FEI Falcon II	Gatan K2 summit

**Fig. 4:**
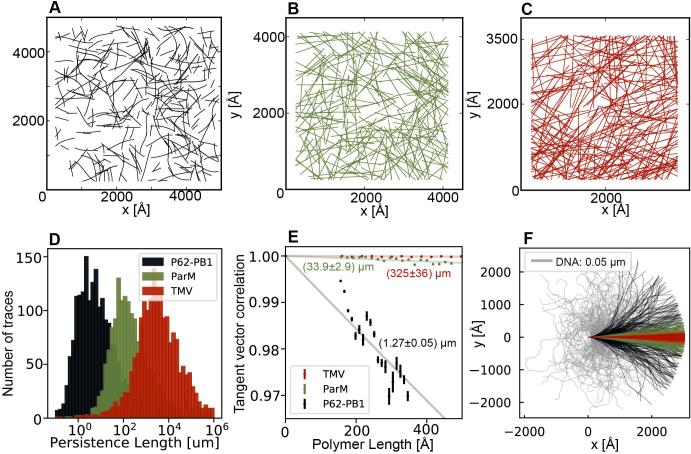
Flexibility analysis of three helical specimens p62-PB1 (black), ParM (green) and TMV (red). (A-C) Projection of 300 determined helix traces of (A) p62-PB1, (B) ParM and (C) TMV onto the area of a micrograph. (D) Histograms of persistence lengths from three different helical specimens, respectively. TMV has the highest persistence length as it is most rigid and PB1-p62 is highly flexible. (E) Persistence length determination of three helical specimens by measuring dot product or tangent vector correlation between adjacent helical segments of different distances. (F) Simulated helical traces plotted on a micrograph for four different populations of specimens with increasing persistence lengths corresponding to DNA (grey), p62-PB1 (black), ParM (green), TMV (red).

For selected 100–150 micrographs from p62-PB1, ParM and TMV data sets, we inspected the shape of the resulting traces (Fig. 4A-C). Based on the coordinates, we computed a persistence length measure for every helix (Eqs. (3), (4), Fig. 4D). The three examples p62-PB1, ParM and TMV show distinct ranges of persistence values, reflecting their difference in stiffness. We estimated the persistence length of the polymer by fitting an exponential decay curve to the correlations of unit tangent vectors along the chain (Fig. 4E). We calculated the following values for the persistence length of p62-PB1 (1.27 ± 0.05 µm), ParM (33.9 ± 2.9 µm) and TMV (325 ± 36 µm), respectively. For visualization of these very different persistence lengths at a scale of a typical micrograph, we simulated a bundle of 100 chains with a length of 3000 Å for each protein polymer (Castro et al., 2015) in addition to DNA (Fig. 4F). The simulation captures the distance of a typical cryo-micrograph and reveals that the ensemble of TMV chains hardly deviates from a straight line, whereas the highly flexible p62-PB1 undergo significant deflection over the same distance.

### 4.2 Performance comparison of automated with interactive tracing methods

**Fig. 5:**
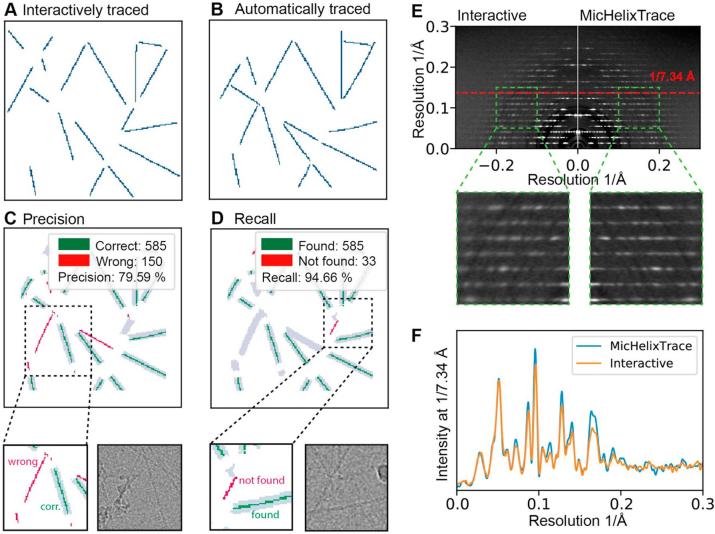
Evaluation of helical tracing performance by determination of precision and recall. (A) Helical traces superimposed on micrograph obtained by interactive helix tracing. Great care was used in the procedure such that the results were considered the ground truth. (B) Helical traces determined by automated tracing procedures MicHelixTrace or RELION. (C) Grey contour areas present an inflated and cropped area of interactively picked helix traces considered ground truth. Automated tracing results were mapped onto grey contours to determine the precision *P*. The overall number of pixels that do or do not coincide with the ground truth can be evaluated to determine true positive and false positive traces and thus the precision *P*. High precision *P* indicates that a large fraction of the found helices correspond to true helices. (D) Grey contour areas here present inflated and cropped automated tracing results. Interactively picked helices (ground truth) were mapped onto grey contours. Pixels that overlap the contour areas were correctly found as true positives, otherwise they are false negatives. High recall *R* means that a high proportion of the ground-truth helices were found. (E) Comparison of averages from in-plane rotated power spectra of TMV obtained from interactively (left) and automatically (right) traced segments by MicHelixTrace. Inset: Magnified area between 0.05 and 0.15 1/Å in y-direction and between 0.1 and 0.2 1/Å in x-direction shows that layer lines are more discrete and less blurred when computed from automatically traced data set due to more precise in-plane angle alignment. (F) Normalized layer line intensity plot 1/7.34 Å confirms that layer line from automated tracing decays slower towards higher resolution compared to interactive tracing.

In order to assess the performance of the here introduced automated MicHelixTrace approach, we compared the results with interactively traced helices using E2HELIXBOXER (Fig. 5A/B). As helices were selected with particular care by the operator, we considered the interactively traced helices as the ground truth. The ratio of helix traces that were overlapping and successfully detected over the “ground truth” helix area corresponds to the precision *P* (Fig. 5C). In turn, the ratio of helix traces of the ground truth over the interactively traced helix area corresponds to the recall *R* and characterizes the sensitivity of detection (Fig. 5D). From a TMV data set consisting of 100 micrographs, using MicHelixTrace we determined high precision and recall values of 0.98 and 0.98, respectively. In order to test the usefulness of these automatically traced viruses, we extracted helical segments, rotated them vertically, averaged the corresponding power spectra and examined them in comparison with interactively picked helices (Fig. 5E). When inspecting a sector of the layer line pattern between 0.05 and 0.15 1/Å resolution, we found the layer line pattern of the MicHelixTrace better defined and less blurred. Closer examination of the layer line at 1/7.34 Å confirms this observation and shows that higher resolution peaks decay faster when interactively cropped helices were segmented and averaged (Fig. 5F). This improvement in the clarity of the power spectra can be attributed to the higher angular accuracy of helix tracing.

### 4.3 Parameter optimization of automated tracing methods using F1-score

**Fig. 6:**
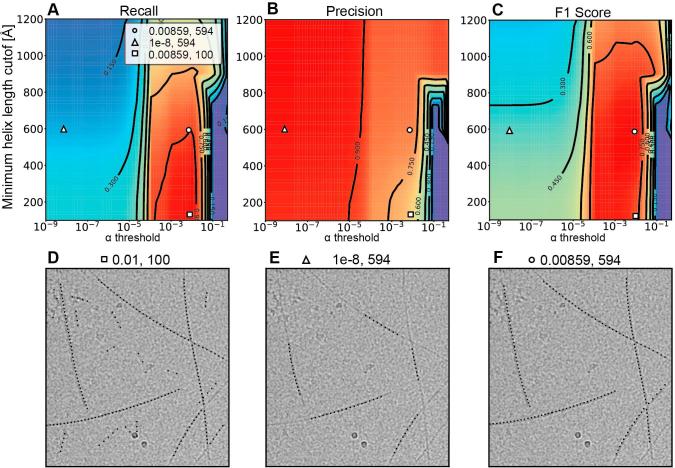
Parameter search for optimal performance of helix tracing. (A–C) Minimum helix length cutoff (y-axis) and α-threshold (x-axis) are being evaluated with respect to (A) recall *R*, (B) precision *P* and (C) F1 score. (D) Choosing a too low minimum helix length cutoff leads to a very high recall *R* with low precision *P* corresponding to promiscuous tracing including contamination and noise. (E) Choosing a too low α-threshold results in very high precision *P* at the expense of poor recall *R*. Using this parameter combination, a large fraction of available helix traces is not detected. (F) Optimal parameter pair yields best performance of the algorithm according to F1-score representing a tradeoff between high precision *P* and high recall *R*.

In order to obtain the coordinates from the cross-correlation map, the steps of thresholding, contamination removal and pruning require additional input parameters to reliably extract helix traces. The input parameters are α-threshold, minimum helix length and the multiple of standard deviation cutoff for persistence length distribution. As the determination of the cross-correlation map constitutes the largest share of the computation time, the extraction steps are relatively fast and can be quickly repeated. In order to achieve optimal results, we found it necessary to adjust the parameters for different helical specimens associated with coordinate extraction. Therefore, we developed an optimization procedure to tune these parameters in a directed manner using the ‘ground truth’ results from a small subset of interactively picked helices. For the three datasets p62-PB1, ParM and TMV, we evaluated recall, precision and F1-score for a combination of the parameter minimum helix length cutoff and α-threshold in 100 Å increments and logarithmic 10-fold increments, respectively. Based on this grid search, we found the best tradeoff between precision and recall in the highest F1-score by the combination of 8.59e-3 and 594 Å α-threshold and minimum helix length cutoff, respectively for ParM filaments (Fig. 6A–C). Superimposing the resulting coordinates on the micrographs confirm the determination of optimal parameters (Fig. 6D–F). To obtain helix traces most reliably, we added an optional parameter optimization step to the MicHelixTrace program using the F1-score for performance comparison with a small subset of interactively traced helices.

### 4.4 Comparison with other automated tracing methods

**Fig. 7:**
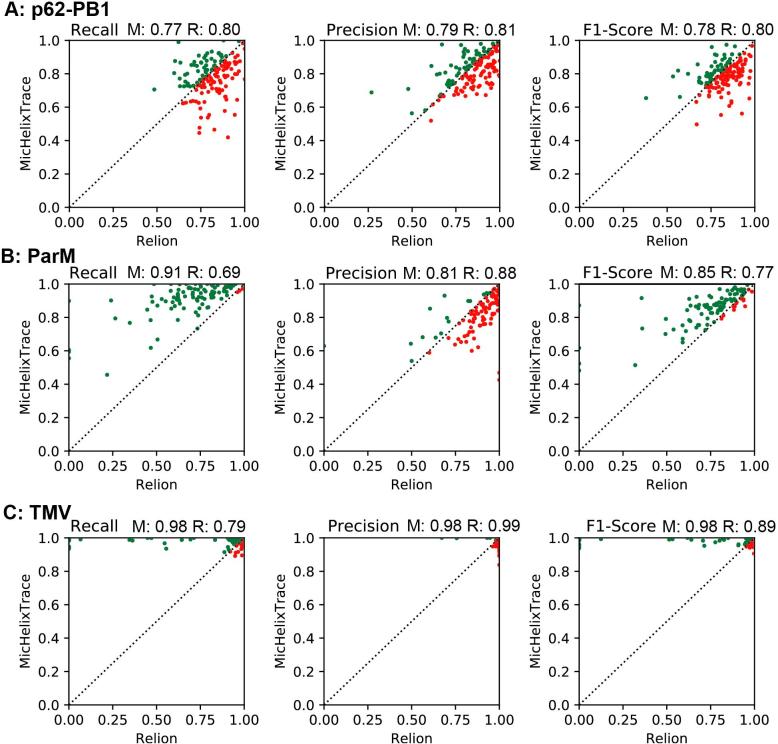
Performance comparison of MicHelixTrace and RELION for automated helical tracing. (A–C) Scatter plot of recall *R* (left), precision *P* (center) and F1-score data pairs (right) obtained by MicHelixTrace (y-axis) and RELION (x-axis) for the same micrograph of (A) P62-PB1, (B) ParM and (C) TMV data sets. The dashed diagonal line presents the case when the same score was obtained by both approaches. For micrographs represented by green dots MicHelixTrace’s scores were higher than RELION’s scores. In turn, for micrographs represented by red dots RELION’s results gave better scores. Average score of micrographs for MicHelixTrace (M) and RELION (R) are documented above the corresponding scatter plot.

In addition to the interactive tracing routine of E2HELIXBOXER, we compared the performance of another available automated tracing algorithm from RELION 2.05. For the three test cases of p62-PB1, ParM and TMV, we evaluated the precision *P* and recall *R* as outlined above and compared RELION and MicHelixTrace results from individual micrographs in scatter plots. The recall *R*, precision *P* and F1-score for flexible p62-PB1 filaments between both methods is almost identical with maximum 3% difference (Fig. 7A). For ParM, MicHelixTrace has a higher recall *R* of 91% instead of 69%, but slightly less precision *P* at 81% instead of 88% when compared with RELION (Fig. 7B). The same trend applies to TMV, where MicHelixTrace has a higher recall *R* of 98% compared with 79% from RELION, at very similar precision *P* (Fig. 7C). Many of the inspected false positives corresponded to some aggregate overlapping with a present helix that were not included by the operator during interactive tracing and thus were not part of the ‘ground truth’ in this analysis.

The MicHelixTrace algorithm has relatively low hardware requirements and is computationally fast. For example, a workstation with 4 Intel i7-880 cores at 3.07 GHz traced 100 micrographs of ParM in just 16 min with 4 GB of memory consumption. RELION is considerably slower as it performs an exhaustive CC search across all angles in five degree increments in 2.8 h. For 148 micrographs of p62-PB1, MicHelixTrace needed 37 min compared with 1.43 h by RELION. For 100 TMV micrographs, RELION took 35 min over 8 min by MicHelixTrace. Both automated approaches are clearly faster than interactive helix tracing, which for the same 100–150 micrographs took 4–5 h depending on the operator. Together, they represent a significant step forward for reducing user interaction in the overall workflow of 3D image reconstruction approaches.

## 5 Discussion

Developing automated tracing tools is critical to study challenging helical assemblies where large amounts of image data are required to optimize sample conditions and to analyze highly flexible specimens. In the current manuscript, we introduced an automated tracing tool that identifies the helical path quickly and reliably by correlating overlapping micrograph tiles with a reference image. These correlation results are accumulated in micrograph maps where only those tiles with locally related in-plane rotations and shifts constructively contribute to a continuous helical path. Random and locally uncorrelated angle and shift distributions correspond to areas free of helical assemblies. Statistical thresholding based on an estimated null distribution enables automated binarization of helical traces. Subsequently, further restraints on minimum and maximum helical path lengths result in localization of helical assemblies. Final pruning is facilitated by straightness analysis of the entire helix population and unrealistically bent helices can be discarded. Along the way, the determination of persistence length yields important material properties of the helical assembly under investigation. Most importantly, the application of automated tracing routines minimizes the time required for initial data analysis, which could for example be exploited in more frequent feedback cycles for sample optimization. Another advantage of such an automated work-flow is that much larger data sets can be analyzed more quickly and smaller subpopulations of straighter specimens can be selected that may be more suitable for high-resolution analysis.

Many principle approaches have been described to detect single particles in electron micrographs (Nicholson and Glaeser, 2001) and more sophisticated approaches are continuously being developed (Wang et al., 2016, Zhu et al., 2017). The principle task of detecting single particles in ice from correlation peaks is more difficult than the detection of continuous traces from elongated helical assemblies as the projected mass of single particles is often much lower than that of filament or virus segments. The prior knowledge on continuity of the object, which is effectively visible as the local correlation of adjacent tiles, imposes a strong restraint that rejects spatially uncorrelated selection of false positive correlation peaks. Finally, larger objects like contamination that still show some degree of spatial correlation can be identified by unexpected length or flexibility measures within the overall population of the assembly. These properties also make the approach sufficiently robust to work with low-resolution features on strongly binned data and neglect more detailed aspects of image formation and still enable precise localization. For example, we demonstrated that angular accuracy of automated tracing is improved when compared with interactive tracing by the human operator. Averages from in-plane rotated power spectra of TMV appear more discrete and less blurred when using segments extracted by coordinates from our proposed automated tracing algorithm (Fig. 5E).

Here, we demonstrate the feasibility of the tracing approach for three very different helical specimens for ParM, p62-PB1 filaments and TMV. The three chosen examples cover helix widths from 70 to 180 Å with TMV having a diameter of 180 Å. We expect that wider helices will be detected as reliably with this tracing procedure as the molecular mass spanning a segment tends to be larger for wider assemblies. If this is the case, the present signal over background in a tile of a wider helix is higher than that of thinner helices and as a result the traces will stand out more clearly from the background within the cross-correlation map. Alongside correctly determined helical paths, a certain percentage of false positive traces are commonly included in the MicHelixTrace results ranging between 2% for rigid TMV to 21% for very flexible p62-PB1 filaments (Fig. 7). Nevertheless, several subsequent processing steps are available that can further remove such false positive traces. First, they can be directly discarded by further 2D classification. Second, during 3D refinement in SPRING stricter straightness criteria on helical segments can be imposed as they are not well alignable and violate smooth helix continuity.

For state-of-the-art automated electron microscopes, datasets commonly consist of thousands of micrographs. Although interactive helix tracing can be achieved with high confidence, it can take days of labor-intense work. Therefore, automated routines such as MicHelixTrace provide a significant time gain. MicHelixTrace is not only suitable to run on a stand-alone quad-core CPU as tested, but it is also able to run on a high-performance computing cluster as micrograph processing is highly parallelizable. Therefore, once distributed to e.g. 100 cores, a ParM dataset of 3000 micrographs can be traced in approximately 20 min providing an enormous time gain over interactive helical tracing. In the future, it is perceivable that such automated algorithms can be employed as live tracing while microscope acquisition advances in particular once initial tracing parameters have been determined (Biyani et al., 2017).

We also compared the performance of MicHelixTrace with the existing tracing routine from RELION 2.05 and found a very similar level of fidelity in the traced results (Fig. 7). Using a small subset of interactively picked helices, we included a parameter optimization routine critical for the extraction of the helix coordinates from the cross-correlation map. In a parameter grid search, we maximize the F1 score as a performance indicator representing a tradeoff between precision *P* and recall *R*. This search is included in MicHelixTrace and determines the combination of parameters that yield optimal tracing results. We found the choice of parameters equally critical for optimal performance of RELION’s tracing routine, which we determined empirically. In addition, we also compared speed of the two procedures. For the presented test cases, MicHelixTrace ran three to ten times faster than the automated tracing routine of RELION on comparable CPUs. In practice, this speed difference can be compensated when the RELION’s tracing is used on GPU based computers (Kimanius et al., 2016).

For certain helical specimens, interactive helix tracing can be challenging as helical traces are often approximated as straight lines. In such cases, the helix has to be subdivided into multiple short stretches and will be treated independently thereby loosing information on the connectivity between line segments for subsequent helical processing. Furthermore, deviation from strict lines provides critical information on the flexibility properties of filamentous and tubular assemblies (Wang et al., 2002). Therefore, based on the traced helix coordinates, we determined the persistence lengths of p62-PB1, ParM and TMV. Cryo-EM images provide a direct way to measure nanoscale material properties from the ensemble of thermally fluctuating molecules (Sachse et al., 2010, Turner et al., 2006). For TMV, the persistence length derived from an alignment and 3D reconstruction procedure has been reported to be 405.2 ± 17.5 µm (Rohou and Grigorieff, 2014), close to our presented estimate of 325 ± 36 µm. For p62-PB1 and ParM, comparative values are not available although ParM exhibits high structural homology to F-actin. For ADP-BeF_3_-bound actin has a persistence length of 13.5 µm (Isambert et al., 1995) and is closely related to our determined ParM value at 33.9 µm. The demonstrated ease of computing the persistence length for a filament might prove useful for analyzing helical assemblies under different conditions. For example, F-actin has been reported to change persistence length when binding to ATP or ADP or also when binding to tropomyosin or troponin (Isambert et al., 1995). The persistence length provides a direct readout of the mechanical properties of the assembly upon temperature increase or in the presence of binding partners. Automated helix tracing combined with flexibility analysis could therefore become a useful tool for sample screening and optimization to assess helix rigidity under different preparation conditions.

Due to the constant ongoing developments in the cryo-EM field, more and more aspects of the structure determination work-flow are being advanced to increase the level of automation in an effort to reduce human intervention. Automated particle detection has been studied as a topic for decades. To date very few robust approaches exist to extract coordinates from filamentous or tubular assemblies of helical symmetries on cryo-micrographs. We here laid out a fast and conceptually simple tile-based reference correlation approach that makes use from restraints derived from the continuity of helical assemblies. We combine the coordinate extraction of helical traces with flexibility analysis. This is particularly useful when considering that most helical assemblies require a minimum degree of straightness to achieve high-resolution resolution reconstructions. Near-atomic resolution structures from two of the three examined examples have been determined. Due to the high persistence length of TMV and high redundancy in helical units, few micrographs are sufficient to generate near-atomic resolution reconstructions (Fromm et al., 2015). Although less rigid, ParM and F-actin filaments have been shown to be suitable for near-atomic resolution structure determination (Bharat et al., 2015b, Ecken et al., 2015). Nevertheless, currently more flexible elongated assemblies remain challenging for high-resolution helical reconstruction (Bednar et al., 1995, Henne et al., 2012). In principle, smaller and smaller segments will compensate for larger deviations from straight assemblies. With foreseen improvements in image quality, using even smaller helical segments it should be possible to reliably align and reconstruct them and thus such flexible helical assemblies can become amenable to high-resolution structure determination.
